# Association of ligamentum teres thickness on MRI with femoral head extrusion in Legg–Calvé–Perthes disease

**DOI:** 10.1007/s00256-026-05349-2

**Published:** 2026-08-24

**Authors:** Felipe Bortoloni Pires Correa, Leonor Garbin Savarese, Marcello Henrique Nogueira-Barbosa

**Affiliations:** Department of Medical Imaging, Hematology and Clinical Oncology, Preto Medical School, University of Sao Paulo, Ribeirão Preto, 14049-09 Brazil

**Keywords:** Round ligament of femur, Magnetic resonance imaging, Legg–Calvé–Perthes disease, Femoral head extrusion, Hip joint

## Abstract

**Objective:**

To evaluate the association between ligamentum teres thickness measured on hip MRI and lateral femoral head extrusion in patients with Legg–Calvé–Perthes disease and to assess the intra- and interobserver reproducibility of these measurements.

**Materials and methods:**

This retrospective study included pelvic radiographs and hip MRI examinations of patients with Legg–Calvé–Perthes disease. Femoral head extrusion was assessed on anteroposterior pelvic radiographs using the Reimers migration index, which served as the reference standard. Two musculoskeletal radiologists independently measured ligamentum teres thickness on MRI in the mediolateral and anteroposterior planes and performed qualitative assessment of ligament morphology. Intra- and interobserver agreement was evaluated using intraclass correlation coefficients and kappa statistics.

**Results:**

Sixty-four hips from 58 patients (mean age, 13.4 years) were included; femoral head extrusion was present in 24 hips (37.5%). Mediolateral ligamentum teres thickness showed excellent intraobserver and good interobserver reproducibility and was significantly greater in hips with extrusion than in those without extrusion (mean, 5.03 mm vs 3.80 mm; *p* = 0.008). Mediolateral thickness correlated positively with the migration index (*ρ* = 0.30, *p* = 0.016). Age was not significantly associated with ligamentum teres thickness or migration index values, and the association between mediolateral thickness and extrusion remained significant after adjustment for age (OR = 1.61; 95% CI, 1.14–2.28; *p* = 0.007). No significant associations were observed for anteroposterior thickness or qualitative ligament morphology.

**Conclusion:**

In Legg–Calvé–Perthes disease, increased mediolateral ligamentum teres thickness on MRI is associated with lateral femoral head extrusion and may represent a reproducible imaging parameter.

## Introduction

Legg–Calvé–Perthes disease is a childhood hip disorder characterized by avascular necrosis of the femoral head, followed by fragmentation, reossification, and remodeling that may result in permanent deformity of the hip joint [1–6]. Although many patients evolve with satisfactory remodeling, a subset develop femoral head deformity, femoroacetabular incongruity, and early degenerative osteoarthritis [7–9]. Consequently, substantial attention has been directed toward identifying imaging findings associated with loss of femoral head containment and worse long-term outcomes.

Among the imaging features associated with unfavorable prognosis, lateral femoral head extrusion is considered one of the most relevant [10–15]. Femoral head extrusion reflects loss of containment within the acetabulum and has been associated with progressive deformity and poorer radiographic outcomes [10, 16]. Several radiographic methods have been proposed to quantify extrusion, including the epiphyseal extrusion index, center–edge angle, acetabulum–head relationship measurements, and the Reimers migration index [10, 13, 17–20]. The Reimers migration index is widely used because of its relative simplicity and reproducibility [19, 20].

The mechanisms underlying femoral head extrusion in Legg–Calvé–Perthes disease remain incompletely understood and are likely multifactorial. Proposed contributors include synovial hypertrophy, cartilage thickening, acetabular remodeling, and alterations in intra-articular soft tissues [16, 21–24]. Among these factors, abnormalities of the ligamentum teres have received relatively limited attention in the MRI literature.

Previous arthrographic and CT arthrographic studies suggested that ligamentum teres enlargement may be associated with femoral head subluxation in Legg–Calvé–Perthes disease [23]. In particular, Kamegaya et al. described swelling of the ligamentum teres in patients with early disease and proposed that this finding could be related to hip subluxation [23]. However, to our knowledge, the relationship between ligamentum teres thickness and femoral head extrusion has not been systematically evaluated using MRI.

Given the increasing use of MRI for the assessment of Legg–Calvé–Perthes disease and the growing interest in intra-articular abnormalities associated with loss of containment, the purpose of this study was to investigate whether ligamentum teres thickness measured on hip MRI is associated with femoral head extrusion assessed on radiographs. A secondary objective was to evaluate the intra- and interobserver reproducibility of ligamentum teres measurements on MRI.

## Materials and methods

This retrospective study was approved by the institutional research ethics committee (CAAE 58814322.1.0000.5440), with a waiver of informed consent. We searched the radiology information system and picture archiving and communication system to identify all patients with a previous diagnosis of Legg–Calvé–Perthes disease sequelae who had both anteroposterior pelvic radiographs and hip MRI examinations performed at our tertiary university hospital between 2004 and 2021. All examinations had been requested for clinical indications, and the present analysis did not interfere with patient management, imaging requests, procedures, or treatment. This study was designed and reported in accordance with the STROBE (Strengthening the Reporting of Observational Studies in Epidemiology) guidelines.

**Fig. 1:**
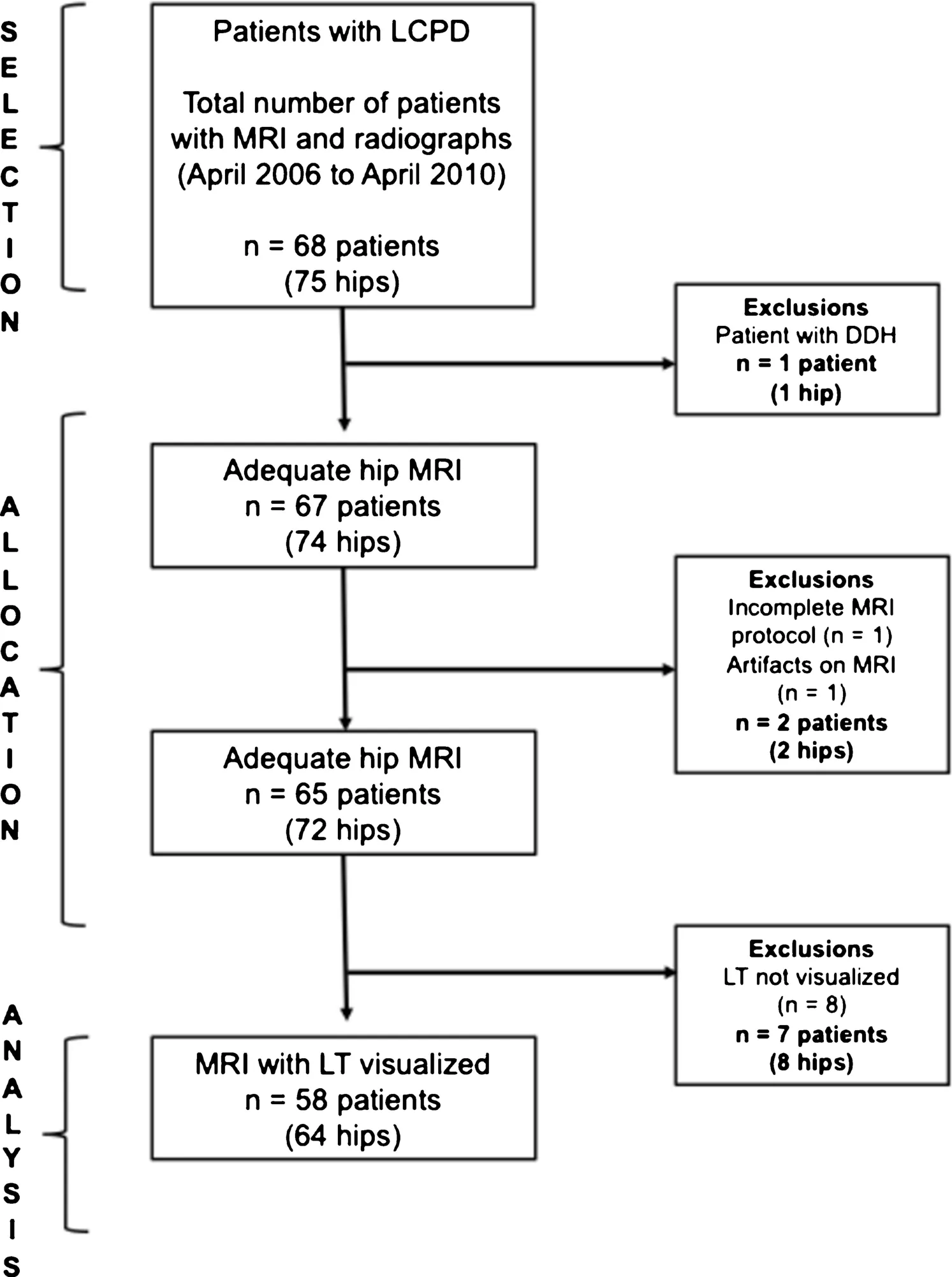
Flowchart showing patient selection and reasons for exclusion. From an initial cohort of 68 patients (75 hips) with Legg–Calvé–Perthes disease and available radiographs and MRI, 58 patients (64 hips) were included in the final analysis

A total of 68 patients were initially identified; seven had bilateral involvement, yielding 75 hips. Exclusion criteria were (1) hip MRI in which the ligamentum teres could not be identified (8 hips), (2) MRI with artifacts that limited ligamentum teres assessment (1 hip), (3) incomplete minimum MRI protocol (1 hip), and (4) another hip disorder associated with Legg–Calvé–Perthes disease, specifically developmental dysplasia of the hip (1 hip). The final sample comprised 58 patients and 64 hips (Fig. 1).

### Image analysis

Image analysis was performed independently by two fellowship-trained musculoskeletal radiologists with 3 years (observer 1) and 9 years (observer 2) of experience, respectively, both staff radiologists at our institution. Independent readings, blinding, and temporally separated sessions were used to minimize consensus bias.

Observers were blinded to patient identity, clinical data, radiographic measurements, migration index values, and each other’s results. Complete blinding to overall hip morphology was not possible because the MRI examinations themselves were being evaluated. Before independent readings, 15 randomly selected cases were reviewed together to standardize criteria and classifications. Observer 1 performed all radiographic measurements and repeated the MRI readings 60 days after the initial session to allow intraobserver reliability assessment.

**Fig. 2:**
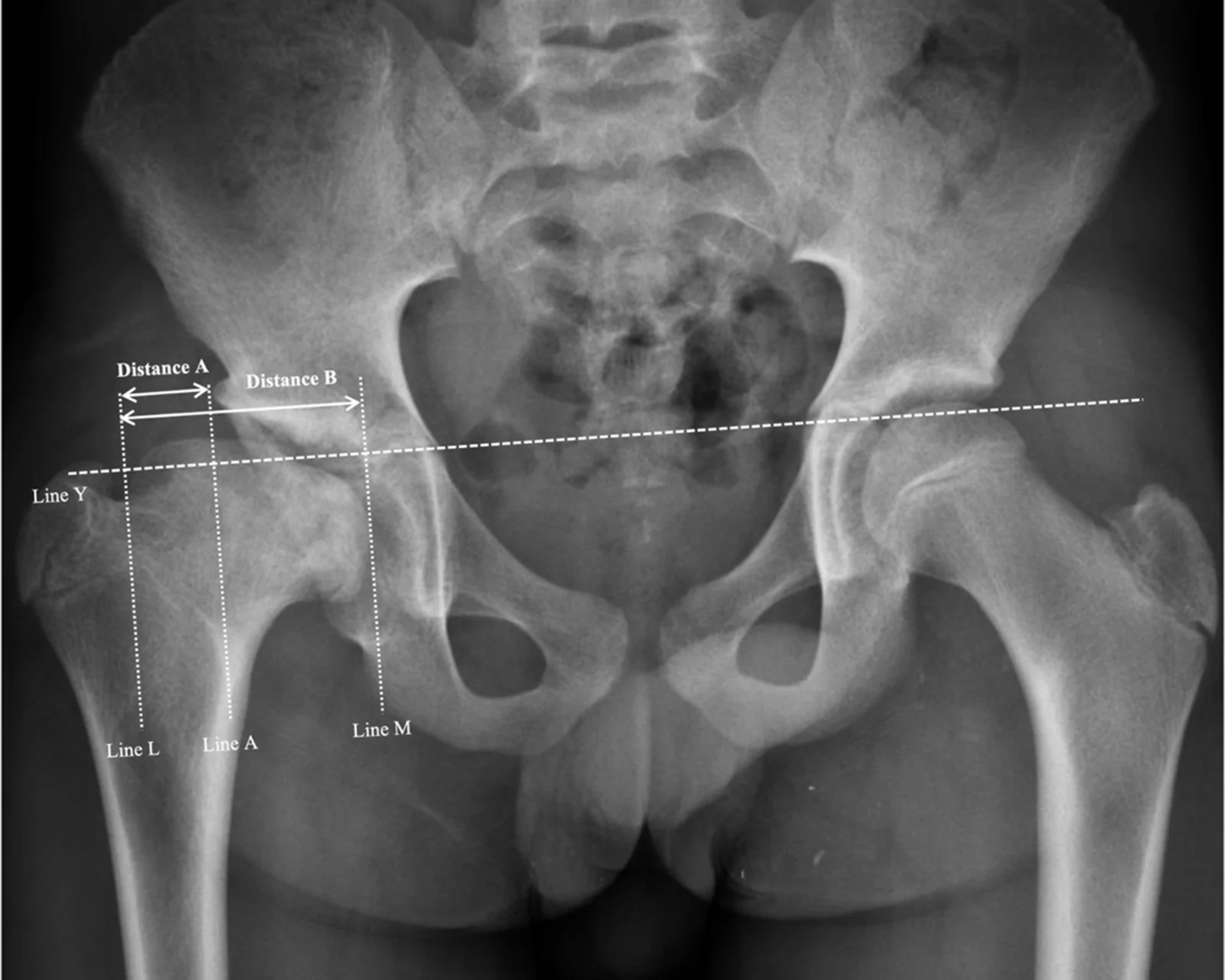
Measurement of the Reimers migration index on an anteroposterior pelvic radiograph. Anteroposterior pelvic radiograph of a 13-year-old boy with Legg–Calvé–Perthes disease involving the right hip. A horizontal reference line (Y-line) is drawn through the triradiate cartilages. Perpendicular lines are positioned at the medial and lateral margins of the femoral head (M-line and L-line, respectively) and at the lateral acetabular margin (A-line). Distance A represents the uncovered lateral portion of the femoral head, whereas distance B represents the total transverse femoral head width. The migration index is calculated as (A/B) × 100. In this patient, the migration index was 38%, indicating presence of femoral head extrusion according to the 20% threshold

All radiographs were anteroposterior pelvic views obtained with adequate positioning criteria (alignment of the coccyx with the pubic symphysis and a distance ≤ 4.0 cm between the tip of the coccyx and the superior border of the symphysis). When more than one radiograph was available for a given patient, the radiograph closest in time to the MRI was selected. Radiographs were used solely to classify hips according to the presence or absence of femoral head extrusion using the Reimers migration index (Fig. 2). The Reimers migration index measured on standardized anteroposterior pelvic radiographs was used as the reference standard for femoral head extrusion.

The migration index was measured as described by Reimers [24]. A horizontal line (Y-line) was drawn through the most distal portions of the iliac bones at the triradiate cartilage, and three perpendicular lines were drawn at the medial and lateral margins of the femoral head (M-line and L-line) and at the lateral acetabular margin (A-line). Distances A and B were obtained, with distance A representing the lateral part of the femoral head not covered by the acetabulum. The migration index was calculated as (A/B) × 100 and expressed as a percentage. Following Green and Joseph [19, 25], a cut-off of 20% was used to dichotomize hips as having extrusion or not; both the dichotomous classification and the continuous migration index values were recorded.

MRI examinations were performed on a 1.5-T scanner (Achieva, Philips Medical Systems, Best, The Netherlands). The minimum protocol for inclusion consisted of coronal short-tau inversion recovery (STIR), axial STIR and/or oblique axial STIR, and coronal T1-weighted sequences of the pelvis or hip, without artifacts impairing assessment of the ligamentum teres. MRI analysis focused on qualitative and quantitative evaluation of the ligamentum teres.

**Fig. 3:**
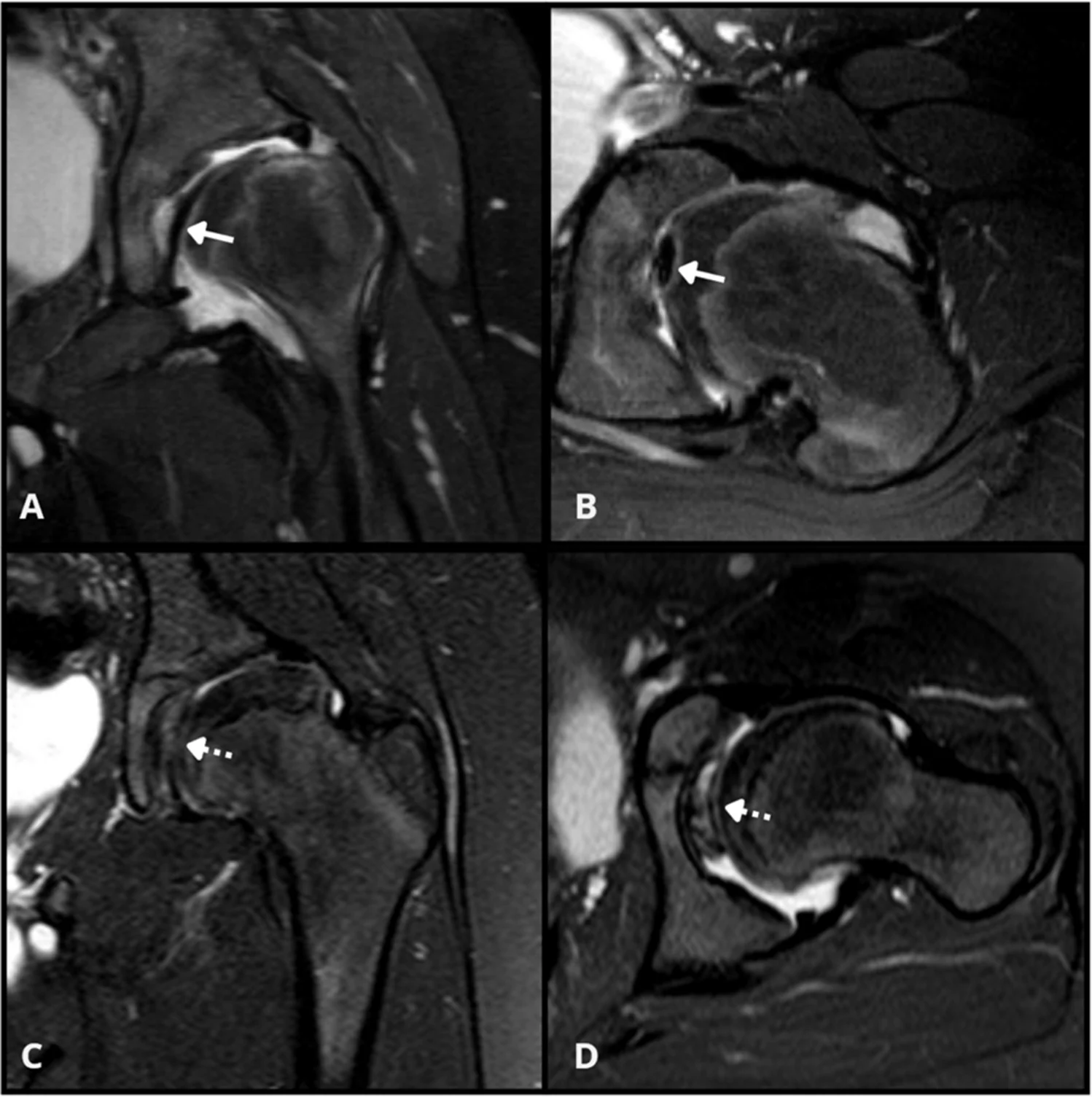
Qualitative classification of the ligamentum teres based on morphology and signal intensity. **A**, **B** Normal ligamentum teres (arrows) on coronal and axial MRI, with sharply defined margins and uniformly low signal intensity. **C**, **D** Abnormal ligamentum teres (dashed arrows) on coronal and axial MRI, showing heterogeneous signal intensity and increased thickness

First, a qualitative classification defined the ligamentum teres as normal or abnormal (Fig. 3). A normal ligament had sharply defined margins and uniformly low signal intensity on all sequences. Ligaments with signal alteration and/or poorly defined margins were classified as abnormal.

**Fig. 4:**
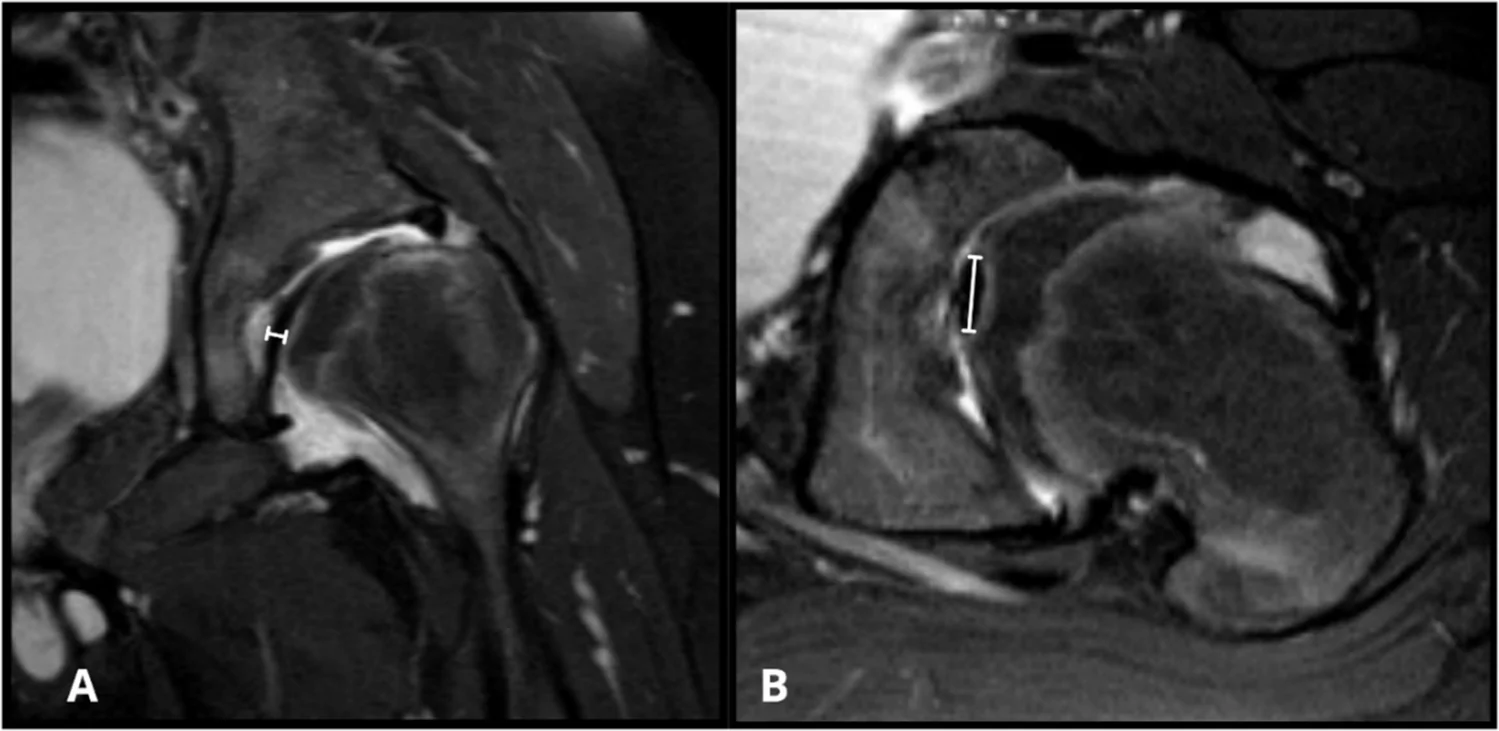
Ligamentum teres thickness measurement on MRI. **A** Coronal fluid-sensitive image showing mediolateral thickness measured at the point of greatest ligament caliber. **B** Axial fluid-sensitive image showing anteroposterior thickness measured at the point of greatest ligament caliber. Calipers indicate the measurement sites

**Fig. 5:**
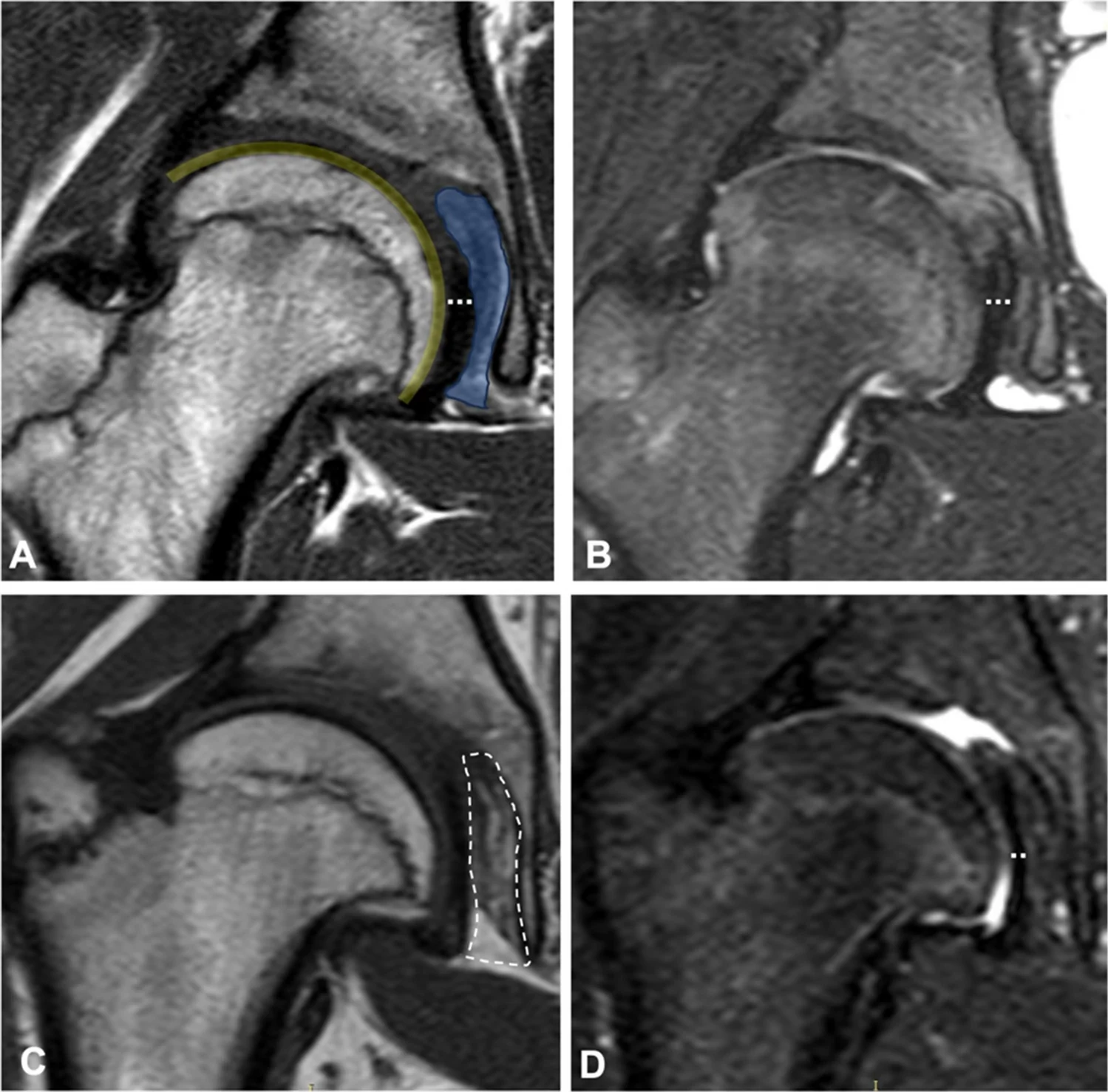
Relevant imaging pitfalls in ligamentum teres measurement. **A, B** Incomplete delineation of the ligamentum teres from the adjacent femoral head cartilage and tissues within the acetabular fossa may result in inaccurate caliper placement. The coronal T1-weighted image (**A**) provides better anatomic distinction between the ligament, the femoral head cartilage (yellow overlay), and the adjacent tissues within the acetabular fossa (blue overlay), guiding subsequent thickness measurement on the corresponding fluid-sensitive image (**B**). **C, D** In another hip, fat within the acetabular fossa is more clearly identified on the T1-weighted image (**C**, dashed contour), facilitating delineation of the true ligament margins and measurement on the corresponding fluid-sensitive image (**D**). Short dashed lines indicate the measurement sites

Quantitative analysis was based on ligamentum teres thickness measurements on coronal and axial or oblique axial fluid-sensitive images (Fig. 4). The coronal measurement was termed the mediolateral thickness and the axial or oblique axial measurement the anteroposterior thickness; both were obtained at the point of greatest caliber. When the ligament margins were difficult to distinguish from the adjacent femoral head cartilage or fat within the acetabular fossa, coronal T1-weighted images were reviewed as an anatomic reference to guide caliper placement on the corresponding fluid-sensitive images (Fig. 5).

The primary objective of the study was to evaluate the association between ligamentum teres thickness on MRI and femoral head extrusion on radiographs. A secondary objective was to assess the intra- and interobserver reproducibility of ligamentum teres thickness measurements.

### Statistical analysis

Normality of continuous variables was assessed using the Shapiro–Wilk test [26]. Normally distributed variables were compared between groups using the independent-samples *t* test with Welch correction when appropriate, whereas nonnormally distributed variables were compared using the Mann–Whitney *U* test. Associations between categorical variables were assessed using the chi-square test or Fisher’s exact test, as appropriate [27].

Correlations between continuous variables were evaluated using Pearson’s correlation coefficient when both variables were normally distributed and Spearman’s rank correlation coefficient when normality assumptions were not met.

To account for the potential confounding effect of age, a logistic regression model was constructed with femoral head extrusion as the dependent variable and mediolateral ligamentum teres thickness and age as independent variables [27].

Intra- and interobserver reproducibility of quantitative ligamentum teres measurements was assessed using single-measure intraclass correlation coefficients (ICCs) [28], interpreted according to Cicchetti [29]. Agreement for qualitative classifications was evaluated using Cohen’s kappa coefficient [30, 31] and interpreted according to Landis and Koch [32]. Statistical significance was defined as *p* < 0.05. All analyses were performed using R software.

## Results

The 58 patients included 12 females (20%) and 46 males (80%). Age at MRI ranged from 6 to 19 years (mean, 13.4 years; SD, 2.9). Time from symptom onset to MRI ranged from 1 to 15 years (mean, 6.8 years; SD, 3.2).

Among the 64 hips evaluated, 24 (37.5%) showed femoral head extrusion on pelvic radiographs, with migration index values ranging from 0 to 38%.

According to observer 1, ligamentum teres morphology was abnormal in 36 hips (56%). Mediolateral thickness ranged from 1.6 to 8.7 mm and anteroposterior thickness from 9.5 to 29.5 mm. According to observer 2, ligamentum teres morphology was abnormal in 41 hips (64%), with mediolateral thickness ranging from 1.5 to 9.0 mm and anteroposterior thickness from 15.0 to 32.0 mm.

Intraobserver agreement for ligamentum teres morphology and thickness (observer 1, two readings) is shown in Table 1. Kappa for the qualitative assessment was 0.58 (95% CI, 0.38–0.78; *p* < 0.001). The intraclass correlation coefficient (ICC) for mediolateral thickness was 0.765 (95% CI, 0.64–0.85; *p* < 0.001) and for anteroposterior thickness was 0.427 (95% CI, 0.19–0.61; *p* < 0.001).

**Table 1:** Intraobserver reliability for qualitative and quantitative assessment of the ligamentum teres on hip MRI

Variable	Coefficient	95% CI	*p* value	Interpretation	Method
Qualitative assessment (normal vs abnormal)	0.58	[0.38; 0.78]	< 0.001	Moderate	Kappa
Mediolateral thickness	0.765	[0.64; 0.85]	< 0.001	Excellent	ICC
Anteroposterior thickness	0.427	[0.19; 0.61]	< 0.001	Fair	ICC

Interobserver agreement between observer 1 (first reading) and observer 2 is summarized in Table 2. Kappa for the qualitative classification was 0.61 (95% CI, 0.42–0.80; *p* < 0.001). The ICC for mediolateral thickness was 0.74 (95% CI, 0.60–0.83; *p* < 0.001), and the ICC for anteroposterior thickness was 0.188 (95% CI, − 0.04 to 0.40; *p* = 0.052).

**Table 2:** Interobserver reliability for qualitative and quantitative assessment of the ligamentum teres on hip MRI

Variable	Coefficient	95% CI	*p* value	Interpretation	Method
Qualitative classification	0.61	[0.42; 0.8]	< 0.001	Substantial	Kappa
Mediolateral thickness	0.74	[0.6; 0.8]	< 0.001	Good	ICC
Anteroposterior thickness	0.188	[−0.04; 0.4]	0.052	Poor	ICC

Age was not significantly correlated with mediolateral ligamentum teres thickness (Pearson *r* = 0.067, *p* = 0.599; Spearman *ρ* = 0.015, *p* = 0.908) or with the migration index (Pearson *r* = 0.062, *p* = 0.626; Spearman *ρ* = 0.069, *p* = 0.589). In addition, age-adjusted logistic regression demonstrated that mediolateral ligamentum teres thickness remained independently associated with femoral head extrusion (OR = 1.61; 95% CI, 1.14–2.28; *p* = 0.007), whereas age was not a significant predictor.

**Fig. 6:**
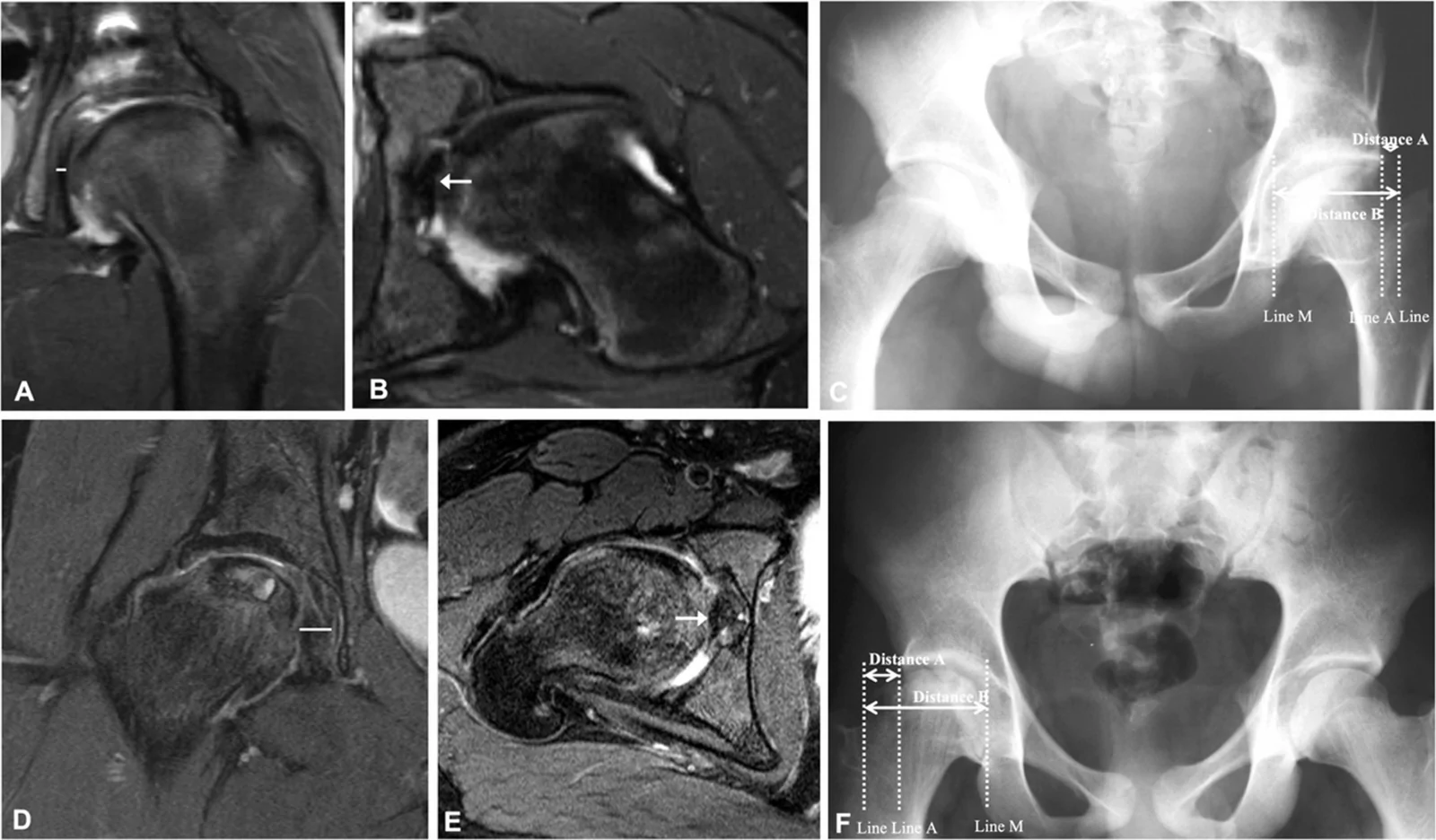
Spectrum of ligamentum teres thickness and associated lateral femoral head migration in Legg–Calvé–Perthes disease. **A**–**C** Sixteen-year-old boy with a thin ligamentum teres. Coronal MRI (**A**) shows a mediolateral ligamentum teres thickness of 3.1 mm, and the corresponding axial image (**B**) identifies the ligament (arrow). The anteroposterior pelvic radiograph (**C**) demonstrates a Reimers migration index of 15%, indicating preserved femoral head containment. **D**–**F** Fourteen-year-old boy with marked ligamentum teres thickening. Coronal MRI (**D**) shows a mediolateral thickness of 9.0 mm, and the corresponding axial image (**E**) identifies the thickened ligament (arrow). The pelvic radiograph (**F**) demonstrates a Reimers migration index of 28%, consistent with lateral femoral head extrusion

**Fig. 7:**
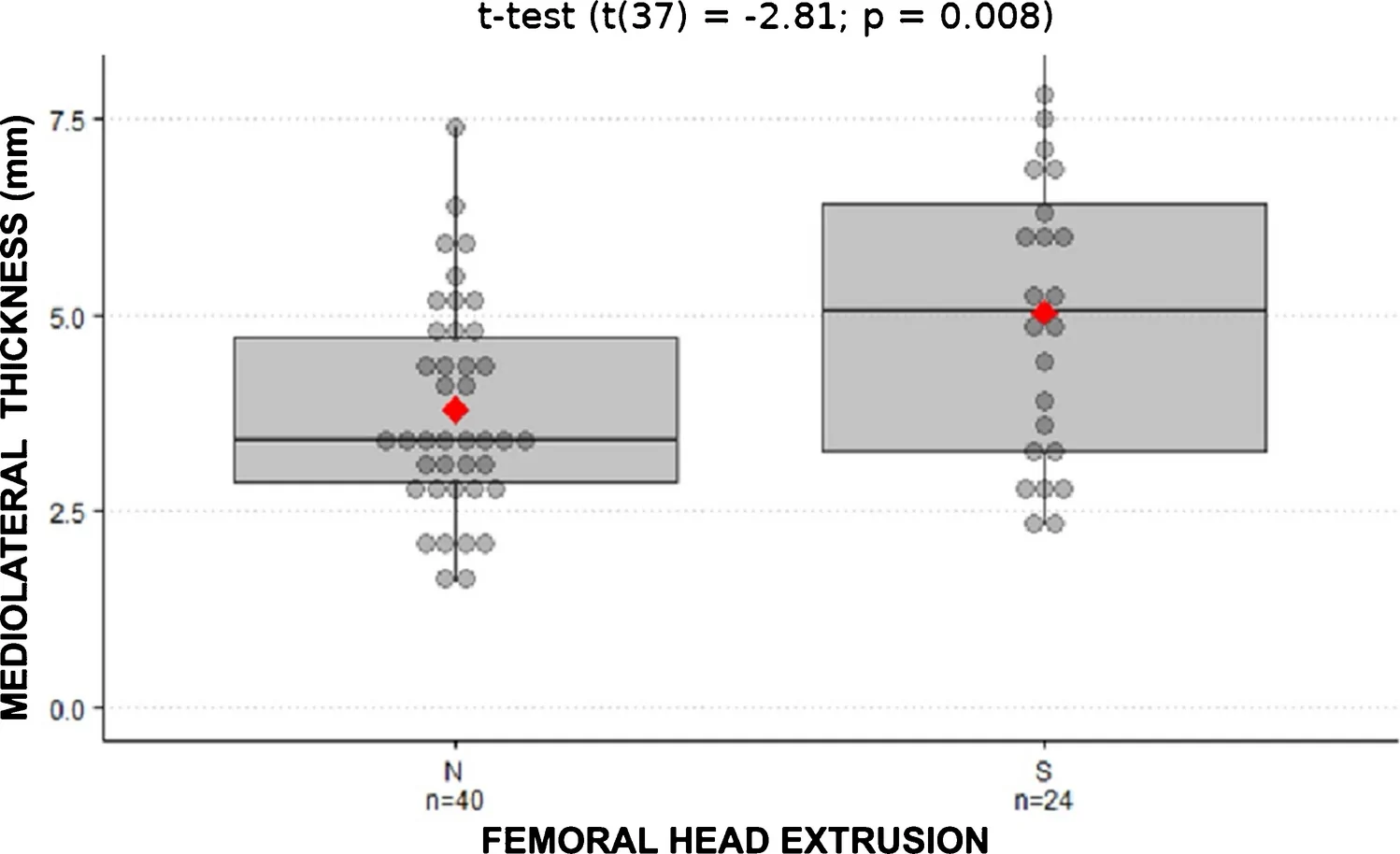
Box-and-whisker plots showing mediolateral ligamentum teres thickness according to the presence of femoral head extrusion. Group N (no extrusion) shows lower mean mediolateral thickness than group S (extrusion), with a statistically significant difference (*p* = 0.008)

The association between ligamentum teres qualitative classification, mediolateral and anteroposterior thickness, and the presence of femoral head extrusion is presented in Table 3. Representative examples of a thin and a markedly thickened ligamentum teres, with corresponding Reimers migration indices of 15% and 28%, respectively, are shown in Fig. 6. A statistically significant difference between hips with and without extrusion was observed only for mediolateral ligamentum teres thickness (mean, 5.03 mm vs 3.80 mm; *p* = 0.008; Fig. 7). No significant associations were found for qualitative classification or anteroposterior thickness.

**Table 3:** Association between ligamentum teres findings and femoral head extrusion

Variable	No extrusion (*n* = 40)	Extrusion (*n* = 24)	*p* value
Altered LT, *n* (%)	19 (47.5)	15 (62.5)	0.603
Mediolateral thickness (mm), mean ± SD	3.8 ± 1.35	5.03 ± 1.89	0.008
Anteroposterior thickness (mm), mean ± SD	17.48 ± 4.63	18.28 ± 4.09	0.472

**Fig. 8:**
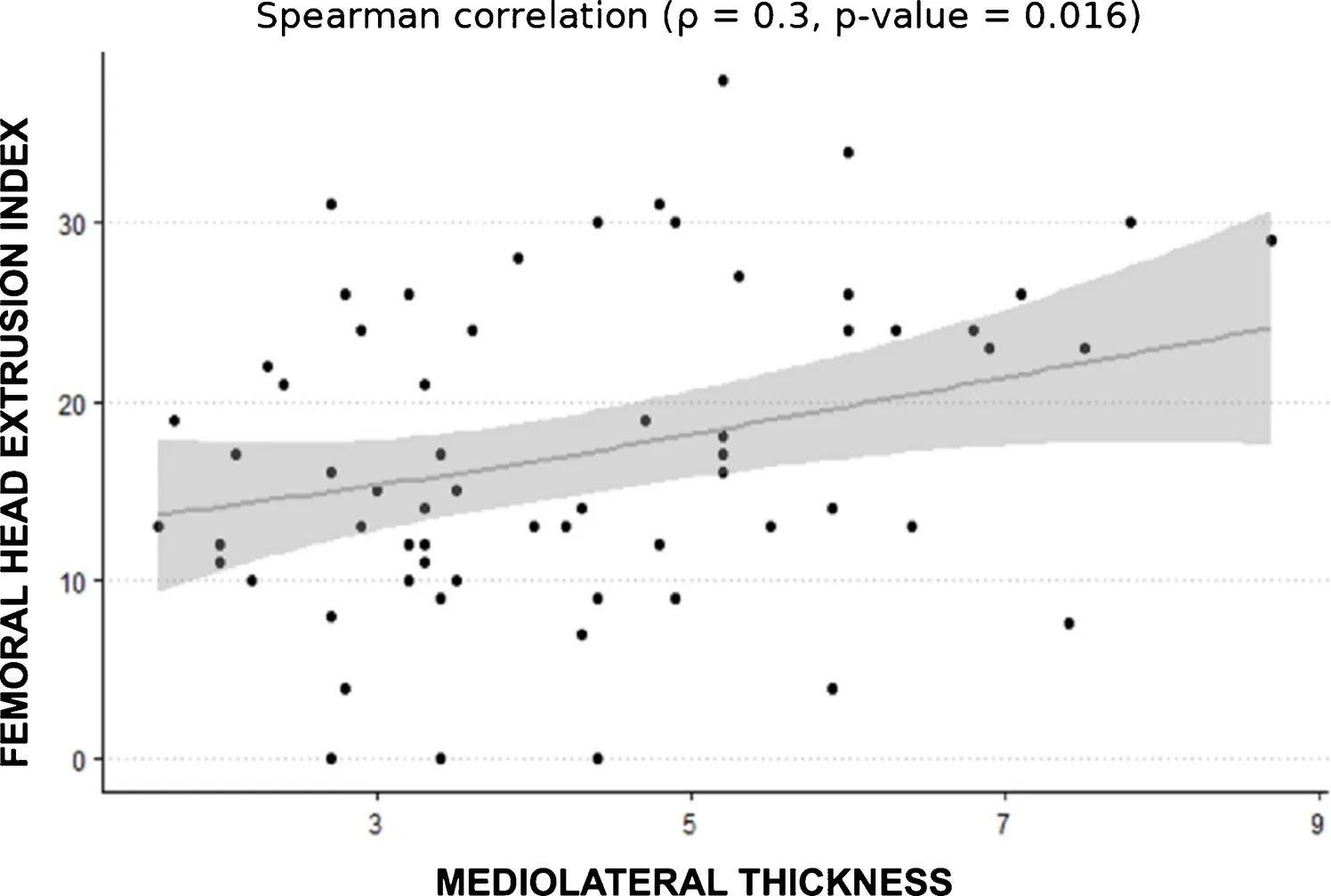
Scatter diagram showing the correlation between mediolateral ligamentum teres thickness on MRI and the femoral head migration index on radiographs. Spearman correlation demonstrates a direct relationship, with higher ligamentum teres thickness associated with higher migration index values (*ρ* = 0.30, *p* = 0.016)

Correlation analysis between ligamentum teres thickness and the continuous migration index values is shown in Table 4. Mediolateral thickness correlated positively with the migration index (*ρ* = 0.30; *p* = 0.016; Fig. 8), whereas anteroposterior thickness did not show a significant correlation (*ρ* = 0.18; *p* = 0.151).

**Table 4:** Correlation between ligamentum teres thickness on MRI and femoral head migration index on radiographs

Variable	Correlation coefficient (*ρ*)	95% CI	*p* value
Mediolateral thickness	0.30	0.09–0.54	0.016
Anteroposterior thickness	0.18	−0.06–0.42	0.151

## Discussion

This study demonstrated an association between ligamentum teres thickening measured on hip MRI and lateral femoral head extrusion assessed on pelvic radiographs in patients with Legg–Calvé–Perthes disease. Mediolateral ligamentum teres thickness was greater in extruded hips than in nonextruded hips and showed a positive correlation with the migration index. In addition, mediolateral thickness measurements showed excellent intraobserver and good interobserver reproducibility, supporting their reliability in clinical and research settings.

These findings demonstrate an association between ligamentum teres thickening and femoral head extrusion in Legg-Calvé-Perthes disease. However, the cross-sectional design does not permit determination of causality, and ligamentum teres thickening may represent either a contributing factor, a secondary adaptation to altered hip biomechanics, or another manifestation of disease progression. Although the difference in mediolateral ligamentum teres thickness between groups was statistically significant, the absolute magnitude of this difference was modest. Therefore, the clinical relevance of this finding remains uncertain and should be interpreted cautiously until validated in studies incorporating clinical outcomes and long-term follow-up.

Kamegaya et al. reported swelling of the ligamentum teres on arthrography and CT arthrography and found a significant association between ligament enlargement and subluxation [23]. In contrast to our study, Kamegaya et al. studied patients with early-onset Perthes disease and concluded that early severe subluxation and consequent incongruity can be attributed to swelling of the ligamentum teres. Therefore, this finding from the previous literature may increase the importance of our finding on magnetic resonance imaging. Our results expand on those observations by demonstrating a similar association using noninvasive MRI and a widely adopted quantitative measure of extrusion, the Reimers migration index [19, 24]. The observed correlation between mediolateral thickness and migration index values suggests that ligamentum teres thickening is associated with the degree of femoral head extrusion, although the temporal and causal relationships remain uncertain.

We did not find a significant association between qualitative ligamentum teres morphology and extrusion. This may reflect the fact that qualitative signal alterations do not necessarily correspond to increased mediolateral thickness; ligaments can show abnormal signal with preserved thickness. Likewise, anteroposterior thickness was not associated with extrusion, which is expected because thickening in this plane does not directly exert lateral mass effect on the femoral head.

Because ligament dimensions may vary according to patient growth, additional analyses were performed to evaluate age as a potential confounder. No significant association was observed between age and ligamentum teres thickness, and the relationship between ligamentum teres thickness and femoral head extrusion remained significant after adjustment for age.

Lateral femoral head extrusion is a well-recognized adverse prognostic factor in Legg–Calvé–Perthes disease and is included in several classification and outcome systems [11, 13, 19, 21–24, 33]. Its etiology has been debated for decades and may involve medial joint space widening due to soft tissue edema in the acetabular fossa, synovial hypertrophy, thickened cartilage, altered acetabular–femoral head relationship as assessed by acetabulum–head indices [34, 35], and changes in the ligamentum teres [19, 20, 36–38]. Jonsäter, using arthrography, described a contrast filling defect medial to the femoral head associated with extrusion and suggested that this defect represented soft tissue edema and ligamentum teres thickening [38]. Kamegaya also reported medial filling defects and confirmed ligamentum teres thickening in a subset evaluated with CT arthrography [23]. Our MRI-based results are in line with these classic arthrographic observations by demonstrating an association between ligamentum teres thickening and femoral head extrusion. Furthermore, mediolateral ligamentum teres thickness showed a positive correlation with migration index values, suggesting that greater ligament thickness tends to occur in hips with more pronounced extrusion. However, because of the cross-sectional design of the study, the temporal and causal relationships between these findings remain uncertain. Ligamentum teres thickening may represent a secondary adaptation to altered hip biomechanics or another manifestation of disease progression.

As emphasized by Joseph et al., the major goal in the management of Legg–Calvé–Perthes disease is to identify the subset of patients at risk for significant deformity and early osteoarthritis and to direct containment strategies accordingly [4, 9, 33]. In this context, reproducible MRI parameters that reflect early or persistent loss of containment may be useful adjuncts to conventional radiographic assessment. The good to excellent reproducibility observed for mediolateral ligamentum teres thickness suggests that this measurement may represent a reproducible imaging marker associated with femoral head extrusion. Whether it provides independent prognostic information beyond established radiographic parameters remains to be determined in longitudinal studies.

This study has limitations. The retrospective design precluded control over disease stage at the time of MRI, and many patients were in advanced stages, which may have influenced the prevalence of synovitis and other early changes. MRI protocols were not fully uniform across the long inclusion period, although a minimum set of sequences was required for inclusion. The cross-sectional nature of the analysis prevents determination of whether ligamentum teres thickening is a cause or a consequence of extrusion. A further limitation is the absence of a healthy control group. Consequently, the present study was designed to compare Perthes hips with and without extrusion rather than to establish normative ligamentum teres thickness values. We also lacked systematic correlation with surgical, arthroscopic, or histopathologic findings, which limits direct validation of MRI-detected ligamentum teres thickening and prevents assessment of its histologic substrate. Prospective longitudinal studies with standardized MRI protocols and multimodal correlation are needed to clarify the temporal relationship between ligamentum teres changes and loss of containment and to assess the prognostic value of this parameter.

In summary, this study shows that ligamentum teres thickening measured on MRI is statistically associated with lateral femoral head extrusion quantified on plain radiographs in patients with Legg–Calvé–Perthes disease. Mediolateral ligamentum teres thickness can be measured with excellent intraobserver and good interobserver reproducibility and may serve as a reproducible imaging parameter associated with femoral head extrusion in patients with this condition.

## Data Availability

Data can be shared on request.
